# Competing Risks Analysis of Cancer-associated Recurrent Thrombosis, Major Bleeds, and Death in a Geriatric Cohort

**DOI:** 10.36469/9822

**Published:** 2015-12-18

**Authors:** Joshua D. Brown, Kelley L. Ratermann, Kelley L. Ratermann, Jeffery C. Talbert, Jeffery C. Talbert, Val R. Adams, Val R. Adams

**Affiliations:** 1 Institute for Pharmaceutical Outcomes and Policy, Lexington, KY; Department of Pharmacy Practice and Science, University of Kentucky College of Pharmacy; Lexington, KY; 2 University of Kentucky College of Pharmacy; Lexington, KY

**Keywords:** venous thromboembolism, deep vein thrombosis, pulmonary embolism, cancer, competing risks, geriatrics

## Abstract

**Background:** Individuals with cancer are at an increased risk of venous thromboembolism (VTE). There is a continued increased risk of recurrent VTE after the initial event as well as increased bleed risk related to VTE treatment.

**Objectives:** This study sought to observe the incidence of recurrent VTE, major bleeding, and death in a geriatric oncology population during treatment for a cancer-associated VTE.

**Methods:** We utilized an insurance claims database of Medicare Advantage beneficiaries 65 and older. The index VTE was identified and individuals were followed up to 180 days to observe an outcome event. Treatment groups were classified among those receiving warfarin, low-molecular weight heparins (LMWH), vena cava (VC) filters with or without anticoagulation, or no treatment. Treatment groups were compared on baseline demographic and clinical characteristics and an inverse probability of treatment weight was used to balance these factors between the groups. A competing risks, time-to-event analysis was performed including treatment only models as well as adjusted models with additional covariates. Causespecific hazards ratios (HRs) and their 95% confidence intervals were reported.

**Results:** Treatment groups differed on baseline variables including age, comorbidities, and tumor sites. After balancing the treatment groups on baseline characteristics, those receiving LMWHs had no difference in recurrent VTE compared to warfarin but had less than half the risk of major bleeding (HR=0.48 [0.27-0.85]). Those receiving VC filters had increased risk of all outcome events relative to warfarin.

**Conclusions:** Patients over the age of 65 with cancer are at a high risk of experiencing recurrent VTE and major bleeding during treatment for a cancer-associated VTE. These results are consistent with United States guidelines which recommend LMWHs over warfarin for treatment and secondary prevention of VTE.

## BACKGROUND

Compared to the general population, individuals with cancer are at 4 to 7 times the risk of developing a venous thromboembolism (VTE).[1-4] Malignancy induces a prothrombotic state which includes activation of the coagulation cascade and is further exacerbated by cancer treatment and surgery.[5] Additional risk factors for VTE in cancer include the site and stage of the tumor, older age, prior history of clots, and comorbidities.[6,7] Although at an already increased risk of death from cancer, VTE carries a substantial risk of mortality with clotting events accounting for up to 10% of all deaths in patients with cancer.[8-10] VTE events, including deep vein thrombosis (DVT) and pulmonary embolism (PE) account for significant lengths of stay and costs in this population with the mean hospital stay ranging from 11 days for DVT and up to 21 days for those with PE.[11]

In the United States, prevention and treatment of VTE in patients with cancer is addressed in American Society of Clinical Oncology guidelines.[12] These guidelines recommend the use of low molecular weight heparins (LMWHs, dalteparin, enoxaparin, tinzaparin) for the initial and long term treatment of VTE for this population. Warfarin is only recommended when LMWHs are contraindicated or limited in use because of cost or other factors including perceived intolerance.[13] In fact, LMWHs have been shown to outperform warfarin in randomized controlled trials and have further benefit in having weight based dosing, fewer drug and food interactions, little monitoring throughout treatment, and maintain positive patient preference despite being an injectable.[8,14-17] However, real-world evidence shows that warfarin is used for a vast majority of cases.[18,19] In addition to anticoagulation therapy, vena cava (VC) filters are commonly utilized in the oncology patient population despite no survival benefit and excess risk compared to other treatment modalities.[20]

Individuals who have had a VTE remain at high risk of experiencing a recurrent VTE event and have high rates of bleeding.[11,21] Recurrent VTE has been reported as high as 21% and bleeding rates as high as 12.4% in cancer patients.[22] Risk factors related to recurrent VTE and adverse bleeding events include tumor site and histology, presence of metastases, age, and certain biomarker or laboratory findings as well as choice of anticoagulant therapy for acute treatment and long-term secondary prophylaxis.[23-26] To our knowledge, no studies have identified risk factors related to recurrent VTE, bleeding, and mortality related to geriatric patients experiencing a cancer-associated VTE. Treatment will be observed for individuals treated with LMWHs, warfarin, VC filters, or who are untreated in a cohort of oncology patients in a large administrative claims database. Demographic and clinical variables associated with each of these three competing outcomes will also be explored.

## MATERIALS AND METHODS

### Data Source and Cohort Identification

This retrospective cohort study used an extract from a large administrative claims database comprised of 1.4 million unique lives with Humana Medicare Advantage medical and pharmacy benefits from 2007 to 2009. The data included inpatient and outpatient medical encounters with procedural codes and diagnoses fields, filled prescription medication claims, and demographic and insurance coverage information linked at the individual level.

The data extract required that an individual have a diagnosis for a malignant neoplasm (104.xx-208.xx) and a DVT (451.xx, 453.xx) or a PE (415.1x) using ICD-9-CM codes for primary diagnosis fields. The earliest date of diagnosis with a DVT or PE was confirmed where at least one claim had a primary diagnosis of DVT or PE and a specific imaging study indicated by diagnostic procedure codes (Appendix).[27] Individuals were excluded if their initial VTE event occurred before their cancer diagnosis or if they were less than 65 years of age at cancer diagnosis. The remaining cohort was required to have at least 180 days of continuous medical and pharmacy coverage during the pre-index period. The 180-day pre-index period was used to assess clinical characteristics including comorbidities and cancer treatment patterns preceding the index event. Lastly, individuals receiving anticoagulant treatment during the 30 days preceding their index event were excluded to ensure that temporality with diagnosis and treatment and to identify treatment naïve patients.

### Cohort Characteristics

Individual demographics and insurance coverage were determined during the pre-index period. Age was categorized 65-69, 70-74, and 75 or older. Race was categorized white, black, and other/unknown. Region was categorized by census regions including South, Midwest, West, and North. Insurance coverage was based on product type (fee-for-service, FFS; health maintenance organization, HMO; or preferred provider organization, PPO).

Tumor site was specified by ICD-9-CM codes including prostate, breast, lung, lymphoma, colon, kidney, pancreas, brain, liver, ovarian, and others. Claims data are limited so that tumor staging is not available. Metastases of the lymph nodes, respiratory, digestive, and other sites were identified using ICD-9-CM codes (195.xx-199.xx). Comorbidities were based on the Charlson Comorbidity Index using the ICD-9-CM coding algorithms by Quan et al. and recorded as a continuous weighted score and categorized by quartiles.[28] Other comorbidities and clinical characteristics were identified by ICD-9-CM codes available in Appendix A and were classified as binary variables.

Medications of interest were identified using Generic Product Identifier (GPI) codes or Healthcare Common Procedure Coding System (HCPCS) codes. Placement of VC filters was identified by procedural codes in the medical claims. VTE treatment choice was determined within the 21 days preceding the index event and was recorded as the last outpatient anticoagulation used to allow for the possibility of bridge therapy or treatment changes. The timing of chemotherapy or radiation therapy was categorized based on its relative timing during the pre-index period to the clotting event as occurring within 30 days, between 31 and 90 days, 91-180 days, or unobserved during the pre-index period using a combination of procedural and medication codes within both the medical and pharmaceutical claims.

### Inverse Probability of Treatment Weight

A multinomial logistic regression model was estimated for treatment choice predicting the probability of each subject to receive warfarin, a LMWH, a VC filter, a VC filter and anticoagulation, or no treatment. All pre-index subject characteristics deemed by the clinical team to be potential predictors of treatment choice as well as related to the outcomes of interest were included. For each subject receiving a particular treatment, the inverse of the probability of receiving that treatment was used to create an inverse probability of treatment weight (IPTW) for each subject. IPTW is a variant of propensity score methods that can be used to weight a regression analysis and has strengths in that no matching or stratification are required thus no reduction in sample size.[29,30] Treatment group comparisons and the performance of the IPTW method were assessed using standardized differences between the groups where a value of >0.10 is considered significant.

### Recurrent VTE, Major Bleeds, and Mortality

Subjects were followed from the index date for up to 180 days or until: 1) they experienced a recurrent VTE; 2) they experienced a major bleed; 3) they died; or 4) they were lost to follow-up due to end of the study period or end of eligibility. The earliest of these events was considered the event of interest. In the case where a death occurred on the same date as one of the other events, that outcome was noted as a death. Recurrent VTE were classified using the same coding algorithm as index event identification and required: a primary diagnosis of a DVT or PE with a specific diagnostic imaging study at least one day after the index event. This was done to help mitigate the chance of the initial event being recoded on a medical management claim as it is unlikely that additional imaging would be required for the index event. Major bleeding events were classified by an algorithm developed by Fang et al. considering a primary diagnosis of intracranial hemorrhage or a bleed requiring a hospitalization or emergency department visit.[31]

A competing risks, time-to-event analysis was performed taking into account the interdependence of the outcome events and producing a cumulative incidence function (CIF) for each outcome. This approach allowed for multivariable analyses with cause-specific coefficient estimates of the predictors for each outcome. Further, competing risks regression allows for the use of the IPTW detailed above so that better direct comparisons could be made between treatment options. We computed the overall CIF for the cohort for each outcome as well as each outcome separately stratified by the treatment received. We fitted a competing risks regression model and included the baseline variables of interest that may still be predictive of outcome events even after IPTW weighting. Hazard ratios and 95% confidence intervals of these final variables are reported.

Data management and analysis was conducted using SAS 9.4 (SAS Institute, Cary, NC) and the manuscript was drafted adhering to the STROBE Statement guidelines for reporting observational studies. The use of de-identified, Humana administrative claims database was approved by the University of Kentucky Institutional Review Board.

## RESULTS

### Characteristics of Treatment Groups

A total of 12,965 subjects met the inclusion and exclusion criteria. Nearly two-thirds of the index events were lower DVTs, 25.6% were PEs, and 8.7% were upper DVTs. Treatment groups, assessed in the acute treatment phase included: 30.4% treated with warfarin, 3.5% treated with LMWHs, 4.1% received a VC filter, 4.4% received a VC filter and anticoagulation, and 57.5% had no observed treatment. Distribution of the index event type was significant between treatment groups with most (82.3%) of the upper DVT index events untreated compared to 60% of lower DVTs and 42.6% of PEs (data not shown). Treatment groups differed significantly across multiple demographic characteristics including age categories, gender, race, region, plan type, and CCI score as well as comorbidities and tumor sites. Baseline demographic and clinical characteristic comparisons between the treatment groups are summarized in Table 1.

### IPTW weighting

An IPTW was calculated for each individual based on the probability of receiving each treatment based on the covariates included in Table 1. The IPTW performed well when used to reweight the population to balance between the covariates. The standardized differences were compared and are shown in Appendix B relative to the warfarin group. Although the IPTW balanced well across all groups, some group-to-group comparisons included significant standardized differences (>0.10) showing the need for some further adjustment in outcome models.

**Table 1.** Baseline Demographic and Clinical Characteristics by Treatment Group

| Characteristic N (%) | Warfarin | LMWH | VC Filter | VC Filter + Anticoagulant | None |
|---|---|---|---|---|---|
| **Total N=12 965** | **3946 (30.4)** | **458 (3.5)** | **536 (4.1)** | **574 (4.4)** | **7451 (57.5)** |
| **Age category*** | | | | | |
| 65-69 | 1038 (26.3) | 176 (38.4) | 124 (22.4) | 166 (28.8) | 2119 (27.9) |
| 70-74 | 1124 (28.4) | 124 (27.1) | 154 (27.9) | 158 (27.4) | 2086 (27.5) |
| 75 and older | 1793 (45.3) | 158 (34.5) | 275 (49.7) | 252 (43.8) | 3392 (44.7) |
| **Gender*** | | | | | |
| Male | 1833 (46.4) | 242 (52.8) | 222 (40.1) | 270 (46.9) | 3917 (51.6) |
| Female | 2122 (53.7) | 216 (47.2) | 331 (59.9) | 306 (53.1) | 3680 (48.4) |
| **Race*** | | | | | |
| White | 2614 (66.1) | 244 (53.3) | 185 (33.5) | 320 (55.6) | 4491 (59.1) |
| Black | 338 (8.6) | 22 (4.8) | 40 (7.2) | 50 (8.7) | 589 (7.8) |
| Other | 1003 (25.4) | 192 (41.9) | 328 (59.3) | 206 (35.8) | 2517 (33.1) |
| **Region*** | | | | | |
| Midwest | 1046 (26.5) | 117 (25.6) | 126 (22.8) | 138 (24.0) | 1593 (21.0) |
| Northeast | 83 (2.1) | 14 (3.1) | 15 (2.7) | 20 (3.5) | 175 (2.3) |
| South | 2421 (61.2) | 289 (63.1) | 368 (66.6) | 369 (64.1) | 5144 (67.7) |
| West | 405 (10.2) | 38 (8.3) | 44 (8.0) | 49 (8.5) | 685 (9.0) |
| **Plan type*** | | | | | |
| FFS | 1898 (48.0) | 214 (46.7) | 200 (36.2) | 219 (38.0) | 2941 (38.7) |
| HMO | 1547 (39.1) | 175 (28.2) | 285 (51.5) | 280 (48.6) | 3615 (47.6) |
| PPO | 510 (12.9) | 69 (15.1) | 68 (12.3) | 77 (13.4) | 1041 (13.7) |
| **CCI score*** | | | | | |
| 0-1 | 1079 (27.3) | 125 (27.3) | 63 (11.4) | 135 (23.4) | 1821 (24.0) |
| 2-3 | 1240 (31.4) | 171 (37.3) | 164 (29.7) | 173 (30.0) | 2349 (30.9) |
| 4-5 | 843 (21.3) | 80 (17.5) | 143 (25.9) | 143 (24.8) | 1593 (21.0) |
| 5+ | 793 (20.1) | 82 (17.9) | 183 (33.1) | 125 (21.7) | 1834 (24.1) |
| **Timing of cancer treatment before index event *** | | | | | |
| >6 months | 3181 (80.4) | 278 (60.7) | 404 (73.1) | 435 (75.5) | 6547 (86.2) |
| 3-6 months | 103 (2.6) | 15 (3.3) | 21 (3.8) | 13 (2.3) | 172 (2.3) |
| 1-3 months | 158 (4.0) | 28 (6.1) | 36 (6.5) | 30 (5.2) | 234 (3.1) |
| <1 month | 513 (13.0) | 137 (29.9) | 92 (16.6) | 98 (17.0) | 644 (8.5) |
| **Initial event*** | | | | | |
| Lower DVT | 2484 (62.8) | 299 (65.3) | 291 (50.5) | 291 (50.5) | 5212 (68.6) |
| Upper DVT | 150 (3.8) | 33 (7.2) | 12 (2.1) | 12 (2.1) | 943 (12.4) |
| PE | 1321 (33.4) | 126 (27.5) | 273 (47.4) | 273 (47.4) | 1442 (19.0) |
| **Comorbidities** | | | | | |
| Leukocytosis* | 181 (4.6) | 21 (4.6) | 55 (10.0) | 39 (6.8) | 421 (5.5) |
| Leukocytopenia* | 40 (1.0) | 14 (3.1) | 8 (1.5) | 5 (0.9) | 100 (1.3) |

*Comparisons between groups significant at $p<0.001$
CCI: Charlson comorbidity index; DVT: deep vein thrombosis; FFS: fee-for-service; HMO: health maintenance organization; LMWH: low molecular weight heparin; PPO: preferred provider organization; PE: pulmonary embolism; VC: vena cava

**Table 1.** Baseline Demographic and Clinical Characteristics by Treatment Group - cont'd

| Characteristic N (%) | Warfarin | LMWH | VC Filter | VC Filter + Anticoagulant | None |
|---|---|---|---|---|---|
| **Total N=12,965** | **3946 (30.4)** | **458 (3.5)** | **536 (4.1)** | **574 (4.4)** | **7451 (57.5)** |
| **Comorbidities** | | | | | |
| Thrombocytosis* | 65 (1.6) | 6 (1.3) | 17 (3.1) | 13 (2.3) | 173 (2.3) |
| Thrombocytopenia* | 177 (4.5) | 25 (5.5) | 37 (6.7) | 30 (5.2) | 450 (5.9) |
| Hypocoagulatory Disorder* | 244 (6.2) | 27 (5.9) | 28 (5.1) | 26 (4.5) | 297 (3.9) |
| Anemia* | 928 (23.5) | 143 (31.2) | 160 (28.9) | 135 (23.4) | 2146 (28.3) |
| Liver disease* | 502 (12.7) | 93 (20.3) | 128 (23.2) | 91 (15.8) | 1055 (13.9) |
| Renal disease* | 854 (21.6) | 90 (19.7) | 195 (35.3) | 144 (25.0) | 1927 (25.4) |
| Hypertension* | 3011 (76.1) | 336 (73.4) | 460 (83.2) | 450 (78.1) | 5892 (77.6) |
| Prior bleed* | 413 (10.4) | 52 (11.4) | 154 (27.9) | 79 (13.7) | 990 (13.0) |
| Obese | 321 (8.1) | 37 (8.1) | 50 (9.0) | 60 (10.4) | 569 (7.5) |
| Myocardial infarction | 399 (10.1) | 50 (10.9) | 66 (11.9) | 65 (11.3) | 891 (11.7) |
| Congestive heart failure* | 954 (24.1) | 81 (17.7) | 144 (26.0) | 111 (19.3) | 1679 (22.1) |
| Peripheral vascular disease* | 896 (22.7) | 99 (21.6) | 159 (28.8) | 141 (24.5) | 2029 (26.7) |
| Cerebrovascular disease* | 731 (18.5) | 60 (13.1) | 174 (31.5) | 129 (22.4) | 1431 (18.8) |
| Dementia* | 110 (2.8) | 6 (1.3) | 35 (6.3) | 17 (3.0) | 245 (3.2) |
| Chronic pulmonary disease* | 1292 (32.7) | 144 (31.4) | 209 (37.8) | 191 (33.2) | 2685 (35.3) |
| Rheumatic disease | 196 (5.0) | 24 (5.2) | 31 (5.6) | 27 (4.7) | 366 (4.8) |
| Peptic ulcer disease* | 96 (2.4) | 11 (2.4) | 38 (6.9) | 15 (2.6) | 256 (3.4) |
| Diabetes w/o complications* | 1277 (32.3) | 153 (33.4) | 211 (38.2) | 193 (33.5) | 2692 (35.4) |
| Diabetes w/ complications* | 436 (11.0) | 43 (9.4) | 78 (14.1) | 63 (10.9) | 1091 (14.4) |
| Paraplegia/hemiplegia* | 103 (2.6) | 10 (2.2) | 56 (10.1) | 29 (5.0) | 207 (2.7) |
| Skin ulcers/cellulitis* | 534 (13.5) | 57 (12.5) | 95 (17.2) | 91 (15.8) | 1186 (15.6) |
| **Tumor site** | | | | | |
| Oral | 67 (1.7) | 10 (2.2) | 14 (2.5) | 13 (2.3) | 153 (2.0) |
| Stomach* | 59 (1.5) | 12 (2.6) | 19 (3.4) | 13 (2.3) | 91 (1.2) |
| Colon* | 321 (8.1) | 54 (11.8) | 46 (8.3) | 61 (10.6) | 562 (7.4) |
| Liver* | 69 (1.7) | 17 (3.7) | 14 (2.5) | 10 (1.7) | 132 (1.7) |
| Pancreas* | 89 (2.3) | 42 (9.2) | 19 (3.4) | 16 (2.8) | 174 (2.3) |
| Lung* | 409 (10.3) | 89 (19.4) | 76 (13.7) | 80 (13.9) | 815 (10.7) |
| Breast | 469 (11.9) | 53 (11.6) | 49 (8.9) | 54 (9.4) | 846 (11.1) |
| Melanoma | 106 (2.7) | 6 (1.3) | 14 (2.5) | 16 (2.8) | 165 (2.2) |
| Uterine* | 73 (1.9) | 18 (3.9) | 14 (2.5) | 27 (4.7) | 120 (1.6) |
| Cervix* | 35 (0.9) | 8 (1.8) | 10 (1.8) | 12 (2.1) | 49 (0.6) |
| Ovarian* | 80 (2.0) | 24 (5.2) | 20 (3.6) | 18 (3.1) | 116 (1.5) |
| Prostate* | 701 (17.7) | 62 (13.5) | 124 (22.4) | 108 (18.8) | 1177 (15.5) |
| Bladder* | 196 (5.0) | 25 (5.5) | 48 (8.7) | 31 (5.4) | 321 (4.2) |
| Kidney* | 110 (2.8) | 16 (3.5) | 28 (5.1) | 22 (3.8) | 230 (3.0) |
| Brain* | 58 (1.5) | 12 (2.6) | 50 (9.0) | 29 (5.0) | 108 (1.4) |
| Thyroid | 21 (0.5) | 1 (0.2) | 5 (0.9) | 5 (0.9) | 62 (0.8) |
| Lymphoma | 335 (8.5) | 52 (11.4) | 55 (10.0) | 47 (8.2) | 628 (8.3) |
| Myeloma | 86 (2.2) | 10 (2.2) | 15 (2.7) | 12 (2.1) | 127 (1.7) |
| Metastatic disease* | 692 (17.5) | 175 (38.2) | 157 (28.4) | 134 (23.3) | 1160 (15.3) |

*Comparisons between groups significant at $p<0.001$

## Competing Risks Analysis

Figure 1 graphs the CIF of each outcome by treatment group. The CIF curves differed significantly across treatment groups and overall group comparisons by Gray's method were significant at $p<0.001$. Three models were estimated including an unweighted, treatment-only model, a IPTW weighted treatment-only model, and an IPTW weighted model including covariates which were not balanced by the IPTW method or that were thought to potentially have residual impact on the outcomes of interest. Table 2 details the outcome-specific HRs and 95% CIs for each treatment group in the treatment-only models with warfarin as the referent treatment group. Some major differences were observed between the unweighted and weighted models further showing some bias in treatment group assignment. In the weighted analysis, those treated with LMWH had similar hazard of experiencing a recurrent VTE and over 50% reduced hazard of experiencing a major bleed (HR 0.48; 95% CI 0.27-0.85). Those receiving a VC filter or a VC filter with anticoagulation were much more likely than the warfarin group to have both recurrent VTE (80-94% increased hazard) and major bleeds (235-492% increased hazard). The untreated group had lower hazard of experiencing a recurrent VTE and no difference in the hazard of experiencing a major bleed. All treatment groups had higher hazards of death but should be interpreted with caution as will be discussed in more detail in the Discussion section.

**Figure 1.** Cumulative Incidence Functions of Outcome Events - Competing risks time-to-event analysis by treatment group over 180 days of follow-up. Cumulative incidence is the percent of the cohort experiencing each event. Cumulative incidence is unweighted and no demographic or clinical characteristics are controlled

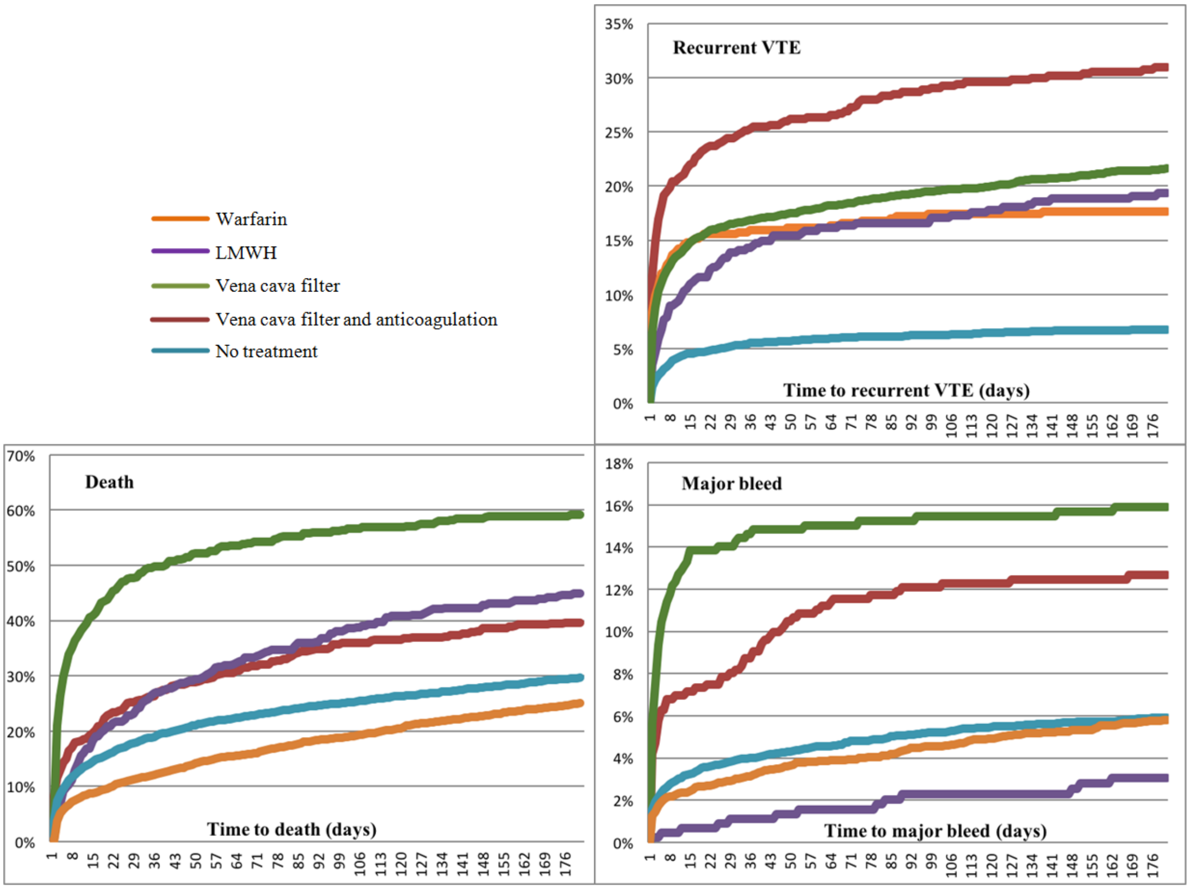

LMWH: low-molecular weight heparin; VTE: venous thromboembolism

**Table 2.** Univariable Treatment Effect on Outcomes

| | | Unweighted | | | Weighted | | |
|---|---|---|---|---|---|---|---|
| | **Treatment** | **HR** | **95%** | **CI** | **HR** | **95%** | **CI** |
| **Recurrent VTE** | Warfarin | **Reference** | | | | | |
| | LMWH | 0.94 | 0.76 | 1.17 | 0.86 | 0.68 | 1.08 |
| | Vena cava filter | 1.41 | 1.14 | 1.74 | 1.79 | 1.48 | 2.17 |
| | Vena cava filter and anticoagulation | 1.90 | 1.62 | 2.23 | 1.94 | 1.65 | 2.28 |
| | None | 0.31 | 0.27 | 0.34 | 0.34 | 0.31 | 0.39 |
| **Major bleeding** | Warfarin | **Reference** | | | | | |
| | LMWH | 0.56 | 0.32 | 0.98 | 0.48 | 0.27 | 0.85 |
| | Vena cava filter | 5.49 | 4.27 | 7.06 | 5.92 | 4.66 | 7.53 |
| | Vena cava filter and anticoagulation | 3.18 | 2.45 | 4.15 | 3.35 | 2.59 | 4.32 |
| | None | 1.00 | 0.85 | 1.18 | 1.05 | 0.89 | 1.24 |
| **Death** | Warfarin | **Reference** | | | | | |
| | LMWH | 2.02 | 1.75 | 2.34 | 1.36 | 1.16 | 1.60 |
| | Vena cava filter | 5.03 | 4.44 | 5.69 | 3.41 | 2.98 | 3.91 |
| | Vena cava filter and anticoagulation | 2.39 | 2.08 | 2.75 | 1.91 | 1.66 | 2.19 |
| | None | 1.15 | 1.07 | 1.24 | 1.16 | 1.08 | 1.25 |

HR: hazard ratio; CI: confidence interval; LMWH: low-molecular weight heparin

Table 3 includes the outcome-specific HRs and 95% CIs for the IPTW weighted model which included additional covariates other than treatment group. Further adjustment for these covariates had marginal effects on the point estimates between the treatment groups. Those with an index PE event had an HR=1.83 (95% CI 1.64-2.03) when compared to lower DVT index events. Individuals who had a history of prior bleeding events during the baseline period had over a 150% increased hazard of major bleed events as well as a 20% increase in recurrent VTE.

**Table 3.** Event Specific Hazard Ratios and 95% Confidence Intervals from IPTW Competing Risks Analysis

| | Recurrent VTE | | | Major Bleed | | | Death | | |
|---|---|---|---|---|---|---|---|---|---|
| | HR | 95% | CI | HR | 95% | CI | HR | 95% | CI |
| **Treatment** | | | | | | | | | |
| Warfarin | | | | | Reference | | | | |
| LMWH | 0.90 | 0.72 | 1.13 | 0.51 | 0.28 | 0.93 | 1.52 | 1.29 | 1.80 |
| VC Filter | 1.85 | 1.53 | 2.24 | 5.70 | 4.42 | 7.36 | 3.45 | 2.94 | 4.04 |
| VC Filter + anticoagulation | 2.01 | 1.71 | 2.37 | 3.47 | 2.68 | 4.50 | 1.80 | 1.53 | 2.12 |
| None | 0.34 | 0.30 | 0.38 | 1.12 | 0.94 | 1.32 | 1.34 | 1.24 | 1.44 |
| **Age Category** | | | | | | | | | |
| 65-69 | | | | | Reference | | | | |
| 70-74 | 0.96 | 0.85 | 1.09 | 0.99 | 0.82 | 1.20 | 1.09 | 1.00 | 1.20 |
| 75 and older | 0.80 | 0.71 | 0.90 | 1.00 | 0.83 | 1.20 | 1.30 | 1.19 | 1.41 |
| **Race** | | | | | | | | | |
| White | | | | | Reference | | | | |
| Black | 1.10 | 0.91 | 1.32 | 1.33 | 1.04 | 1.70 | 1.32 | 1.15 | 1.52 |
| Other | 1.04 | 0.68 | 1.60 | 0.98 | 0.51 | 1.86 | 0.97 | 0.68 | 1.38 |
| **CCI Score** | | | | | | | | | |
| 0-1 | | | | | Reference | | | | |
| 2-3 | 1.10 | 0.94 | 1.29 | 1.32 | 1.02 | 1.72 | 1.22 | 1.08 | 1.37 |
| 4-5 | 1.03 | 0.82 | 1.31 | 1.46 | 1.05 | 2.02 | 1.39 | 1.20 | 1.62 |
| 5+ | 1.23 | 0.86 | 1.77 | 1.30 | 0.83 | 2.03 | 1.48 | 1.19 | 1.83 |
| **Timing of cancer treatment before index event** | | | | | | | | | |
| >6 months | | | | | Reference | | | | |
| 3-6 months | 0.73 | 0.49 | 1.07 | 0.97 | 0.61 | 1.57 | 1.27 | 1.06 | 1.52 |
| 1-3 months | 0.82 | 0.59 | 1.12 | 0.93 | 0.62 | 1.39 | 1.32 | 1.14 | 1.53 |
| <1 month | 0.87 | 0.71 | 1.06 | 0.94 | 0.70 | 1.26 | 1.27 | 1.15 | 1.40 |
| **Index Event** | | | | | | | | | |
| Lower DVT | | | | | Reference | | | | |
| Upper DVT | 0.86 | 0.71 | 1.04 | 0.81 | 0.63 | 1.05 | 0.74 | 0.64 | 0.85 |
| Pulmonary embolism | 1.83 | 1.64 | 2.03 | 1.16 | 0.98 | 1.37 | 1.65 | 1.54 | 1.78 |
| **Comorbidities** | | | | | | | | | |
| Leukocytosis | 1.18 | 0.94 | 1.47 | 1.62 | 1.26 | 2.08 | 1.36 | 1.21 | 1.53 |
| Leukocytopenia | 0.95 | 0.58 | 1.57 | 1.39 | 0.80 | 2.43 | 1.10 | 0.86 | 1.40 |
| Thrombocytosis | 1.08 | 0.85 | 1.38 | 1.07 | 0.80 | 1.42 | 1.12 | 0.98 | 1.27 |
| Thrombocytopenia | 1.07 | 0.75 | 1.54 | 0.89 | 0.54 | 1.46 | 1.08 | 0.87 | 1.34 |
| Hypocoagualotory disorder | 0.97 | 0.44 | 2.13 | 0.66 | 0.22 | 2.05 | 2.05 | 1.40 | 3.00 |
| Anemia | 0.35 | 0.30 | 0.41 | 0.77 | 0.65 | 0.91 | 0.97 | 0.90 | 1.04 |
| Liver disease | 1.09 | 0.92 | 1.28 | 0.96 | 0.77 | 1.19 | 1.15 | 1.06 | 1.26 |
| Renal disease | 1.14 | 0.98 | 1.33 | 1.22 | 1.01 | 1.48 | 1.24 | 1.14 | 1.36 |
| Hypertension | 1.15 | 1.00 | 1.32 | 0.94 | 0.76 | 1.16 | 0.85 | 0.77 | 0.93 |

HR: hazard ratio; CI: confidence interval; LMWH: low molecular weight heparin; VTE: venous thromboembolism; DVT: deep vein thrombosis; VC: vena cava; CCI: Charlson comorbidity index; IPTW: inverse probability of treatment weight

**Table 3.** Event-specific HRs and 95% CIs from IPTW Competing Risks Analysis - cont'd

| | Recurrent VTE | | | Major Bleed | | | Death | | |
|---|---|---|---|---|---|---|---|---|---|
| | HR | 95% | CI | HR | 95% | CI | HR | 95% | CI |
| **Comorbidities - cont'd** | | | | | | | | | |
| Prior bleed | 1.19 | 1.01 | 1.41 | 2.53 | 2.10 | 3.04 | 1.20 | 1.08 | 1.33 |
| Obese | 1.21 | 1.02 | 1.43 | 1.13 | 0.89 | 1.44 | 0.96 | 0.84 | 1.09 |
| Myocardial infarction | 0.90 | 0.75 | 1.08 | 1.11 | 0.90 | 1.36 | 0.93 | 0.84 | 1.04 |
| Congestive heart failure | 0.85 | 0.74 | 0.98 | 1.18 | 0.98 | 1.40 | 1.23 | 1.13 | 1.34 |
| Peripheral vascular disease | 1.02 | 0.90 | 1.16 | 1.01 | 0.85 | 1.20 | 0.92 | 0.85 | 1.00 |
| Cerebrovascular disease | 0.86 | 0.74 | 0.99 | 1.26 | 1.06 | 1.49 | 1.01 | 0.93 | 1.10 |
| Dementia | 0.85 | 0.62 | 1.19 | 0.68 | 0.43 | 1.07 | 1.38 | 1.17 | 1.63 |
| Chronic pulmonary disease | 1.02 | 0.90 | 1.15 | 1.05 | 0.89 | 1.24 | 1.22 | 1.13 | 1.32 |
| Rheumatic disease | 1.16 | 0.93 | 1.45 | 1.24 | 0.94 | 1.64 | 1.07 | 0.92 | 1.24 |
| Peptic ulcer disease | 0.83 | 0.58 | 1.18 | 0.96 | 0.70 | 1.32 | 1.01 | 0.83 | 1.22 |
| Diabetes w/o complications | 0.88 | 0.77 | 1.00 | 1.10 | 0.92 | 1.30 | 0.97 | 0.90 | 1.06 |
| Diabetes w/ complications | 0.89 | 0.72 | 1.10 | 1.03 | 0.81 | 1.31 | 1.00 | 0.88 | 1.12 |
| Paraplegia/Hemiplegia | 1.12 | 0.84 | 1.51 | 1.61 | 1.18 | 2.20 | 1.47 | 1.24 | 1.73 |
| Skin ulcers/Cellulitis | 0.85 | 0.74 | 0.98 | 0.89 | 0.72 | 1.10 | 1.00 | 0.90 | 1.10 |
| **Tumor Site** | | | | | | | | | |
| Oral | 0.68 | 0.43 | 1.06 | 1.20 | 0.74 | 1.94 | 1.05 | 0.87 | 1.27 |
| Stomach | 1.66 | 1.14 | 2.42 | 0.57 | 0.25 | 1.29 | 1.39 | 1.15 | 1.69 |
| Colon | 0.99 | 0.81 | 1.20 | 1.17 | 0.90 | 1.52 | 1.07 | 0.96 | 1.19 |
| Liver | 0.74 | 0.45 | 1.21 | 0.48 | 0.21 | 1.08 | 1.09 | 0.91 | 1.29 |
| Pancreas | 0.67 | 0.43 | 1.04 | 1.09 | 0.66 | 1.81 | 1.64 | 1.41 | 1.89 |
| Lung | 0.72 | 0.59 | 0.88 | 1.14 | 0.87 | 1.49 | 1.27 | 1.16 | 1.40 |
| Breast | 1.02 | 0.87 | 1.20 | 0.98 | 0.75 | 1.28 | 0.88 | 0.78 | 0.99 |
| Melanoma | 0.86 | 0.61 | 1.23 | 0.99 | 0.60 | 1.63 | 0.88 | 0.73 | 1.07 |
| Uterus | 0.75 | 0.49 | 1.13 | 0.60 | 0.28 | 1.26 | 1.19 | 0.95 | 1.50 |
| Cervix | 1.03 | 0.56 | 1.88 | 1.47 | 0.64 | 3.37 | 1.29 | 0.94 | 1.77 |
| Ovarian | 0.89 | 0.57 | 1.39 | 1.10 | 0.55 | 2.19 | 1.24 | 1.02 | 1.51 |
| Prostate | 0.68 | 0.43 | 1.06 | 1.14 | 0.93 | 1.40 | 0.88 | 0.80 | 0.98 |
| Testicular | 1.66 | 1.14 | 2.42 | - | - | - | 1.02 | 0.70 | 1.47 |
| Bladder | 0.99 | 0.81 | 1.20 | 1.37 | 1.02 | 1.84 | 1.24 | 1.08 | 1.42 |
| Kidney | 0.74 | 0.45 | 1.21 | 1.10 | 0.75 | 1.62 | 1.04 | 0.88 | 1.22 |
| Brain | 0.67 | 0.43 | 1.04 | 0.81 | 0.46 | 1.43 | 1.32 | 1.10 | 1.60 |
| Thyroid | 0.72 | 0.59 | 0.88 | 2.17 | 1.19 | 3.98 | 0.80 | 0.50 | 1.26 |
| Lymphoma | 1.02 | 0.87 | 1.20 | 0.97 | 0.71 | 1.32 | 1.25 | 1.11 | 1.40 |
| Myeloma | 0.86 | 0.61 | 1.23 | 1.56 | 1.02 | 2.40 | 0.91 | 0.72 | 1.15 |
| Metastatic disease | 1.14 | 0.24 | 5.37 | 0.60 | 0.46 | 0.79 | 1.85 | 1.69 | 2.03 |

HR: hazard ratio; CI: confidence interval; LMWH: low molecular weight heparin; VTE: venous thromboembolism; DVT: deep vein thrombosis; VC: vena cava; CCI: Charlson comorbidity index; IPTW: inverse probability of treatment weight

## DISCUSSION

This study is the first study to our knowledge to assess the incidence of outcome events after an initial cancer-associated VTE in a geriatric oncology population. This population is of particular interest given the increased risk of treatment related complications as well as a high baseline risk of mortality from multiple causes.

Our findings are consistent with previous studies showing a high rate of recurrent VTE and major bleeding after an index VTE which differed across the treatment modality.[18,19,22,23,32] After balancing the treatment groups on baseline characteristics, we found no differences between warfarin and LMWHs and risk of recurrent VTE but showed that warfarin treated patients had more than twice the hazard of a major bleed. Those receiving a filter had very large increases in all outcome events relative to both LMWH and warfarin. This generally confirms the recommendations made by U.S. clinical practice guidelines which prefer LMWHs over warfarin and only recommend VC filters when other treatments are contraindicated.[33]

LMWHs have been shown to have a large incremental cost-effectiveness ratio compared to warfarin[34] as well as perceived patient intolerance and higher pharmacy costs.[13] Nevertheless, oral anticoagulation with warfarin can be difficult in practice given the patient variability in dosing and required monitoring as well as potential drug-drug interactions with chemotherapy, changes in body weight, altered liver or renal function, and unpredictable gastrointestinal absorption due to vomiting or mucositis.[33] These considerations will become more important as novel oral anticoagulants (NOACs; apixaban, rivaroxaban, dabigatran, edoxaban) are beginning to see use in this population and are currently being investigated for efficacy and safety for primary and secondary prophylaxis of cancer-associated VTE.[35-40]

There is likely significant bias in the choice of treatment for a given individual and is shown in our baseline comparisons. Using the IPTW in the regression analysis helps to balance these differences in a way analogous to the randomization process of a randomized controlled trial. However, the IPTW is limited to the logistic regression model specification and may not capture all the bias that is present. We included several demographic and clinical characteristics which could drive the choice of treatment in this population. Important factors which could not be controlled for given the nature of claims data include tumor staging and histology as well as other important clinical history that may contribute to treatment choice or baseline risk of outcome events. However, our study is strengthened by a large sample size which includes some relatively rare cancers, such as multiple myeloma, brain, and renal cancers, that have lacked investigation in previous studies.[7,41]

Of particular interest throughout the conduct of this study is the untreated group which comprised the majority of the identified cohort. This finding is not unique to our study with a recent study of real-world data in another population in the United States reporting a treatment rate of 50%.[35,42,43] We hypothesize that this group could consist of several unique profiles of individuals. For one, a proportion of this group may include those that are unfit to receive any treatment given a poor prognosis related to the underlying cancer or the index event. There may also be cases which the index VTE event was considered asymptomatic or not a high priority for treatment based on unobservable patient factors. Further, there may have been some false positive misclassification of index events which met the coding criteria. However, as discussed below in the limitations, the sensitivity and specificity of the algorithm is expected to be >90% given the high risk of events in this population.[27] It would further be expected that misclassification would not necessarily differ between treatment groups and would be evenly distributed among the treatment groups. While some groups have used treatment as confirmation of index VTE events,[44] the inclusion in our study would not impact the direct comparisons made between the other treatment groups and was considered a more thorough analysis.

Lastly, it is possible that other, non-guideline recommended treatments were used or that medications were purchased out-of-pocket or using another insurance benefit. Anywhere from 10-20% of warfarin prescription are purchased through low-cost generic programs in the United States and may contribute to exposure misclassification in this population.[45,46]

**Limitations**

Our study is subject to several limitations inherent to claims-based studies.[47,48] We relied on ICD-9-CM coding available in the claims to diagnose study subjects in addition to requiring the presence of specific imaging modalities to confirm index and recurrent VTE events. It is impossible to confirm a positive diagnosis using these data; however, claims-based coding algorithms for VTE have been shown to perform strongly especially when there is a high risk of VTE in the population.[49,50] In addition to this validated algorithm, we further required the presence of an imaging procedure specific for diagnosis of VTE events to indicate an event of interest which will have likely increased the specificity of our coding algorithm and insured that a recurrent VTE event was a new event and not management of the previous index event. Further, we considered treatment group assignments based on the pattern of medication use or procedural codes within the first 10 days of the index events and held the treatment group assignment throughout the 180 days follow-up. Realistically, treatment, as well as other factors, may change drastically over the course of the study period. However, the majority of outcome events occurred during the first 30 days post-index where treatment choice and individual factors would generally remain stable. Future work should identify and account for important factors that may vary over time for inclusion in analytic models.

We used a competing risks framework given that the outcome events cannot be considered independent of each other, i.e. experiencing one may preclude experiencing another or one event may cause another. Failure to do so can overestimate survival for traditional Kaplan-Meier based analyses.[41] In this population especially, the competing risk of death is a contribution by many factors including the advanced age of the cohort, having cancer, as well as the risk of death from the other outcome events.[41] Given the nature of the data, we could not assign cause of death in this study. For example, if death was caused by a major bleed or recurrent VTE but not submitted for claims adjudication, the alternative outcome would not be observed. Thus, the findings related to the death should be interpreted with caution. Lastly, our results are from a commercially insured population of individuals with Medicare Advantage plans over the age of 65. Thus, our results may not be generalizable to the general geriatric population but do provide insight into the burden of these outcome events in this population which makes of about 30% of those with Medicare insurance in the United States.[51]

## CONCLUSION

There is a high rate of recurrent VTE and major bleeding events within 180 days of a cancer-associated VTE. The risk of experiencing these outcomes varied across treatment groups showing no difference between warfarin and LMWHs for recurrent VTE but twice the risk of major bleeding with warfarin. Patients receiving filters were at largely increased risk of all outcome events. These findings are consistent with U.S. clinical practice guidelines which prefer LMWH over warfarin in both the acute and long-term treatment after a cancer-associated VTE and only recommend vena cava filters if other treatments are contraindicated.

## CONFLICT OF INTEREST DECLARATION

This study was not funded, and the authors have no conflict of interest to disclose.

## APPENDICES

**Appendix A:** Coding algorithms

*Comorbidities not included in Charlson comorbidity index (ICD-9-CM codes):*

<u>Hypocoagulation defects:</u> 2860, 2861, 2862, 2863, 2864, 2865, 28652, 28653, 28659, 2866, 2867, 2869
<u>Other coagulation/hemorrhagic:</u> 2870, 2871, 2872, 2878, 2879, 7827
<u>Thrombocytopenia:</u> 2873, 2874, 2875
<u>Decreased white cell count:</u> 2885
<u>Elevated white cell count:</u> 2886
<u>Hypercoagulation (primary or secondary):</u> 28981, 28982
<u>Anemia:</u> 280-285

| DVT/PE Coding Algorithm | |
|---|---|
| Deep vein thrombosis 451, 452, 453 | ICD-9-CM: Lower DVT - 451.11, 451.19, 451.81, 453.4, 453.41, 453.42; Upper DVT - 451.2, 451.9, 453.1, 453.2, 453.8, 453.9 |
| Pulmonary embolism | ICD-9-CM: 415.1x |
| Imaging studies | CPT codes: 93306, 93307, 93308, 93325, 93312, 93313, |
| Echocardiography | 93314, 93318, 93320, 93321, 93325, 76881, 76882, |
| Chest X-Ray | 93970, 93971, 93975, 93976, 75820, 75822 |
| V/Q Scan | CPT: 71020 CPT: 78585 |
| CT scan/CT Angiography | CPT: 71275, 21250, 71260, 71270, 73200, 73201, 73202, |
| MRI/MRI Angiography | 73700, 73701, 73702, 73206, 73706 |
| Ultrasound | CPT: 71555, 73218, 73220, 73718, 73720, 73225, 73725, 75820, 75822, 76882, 93970, 93971, 93975, 93976 |

CPT: Current Procedural Terminology; CT: computed tomography; DVT: deep vein thrombosis; ICD-9-CM: International Classification of Diseases, 9th Revision, Clinical Modification; MRI: magnetic resonance imaging; PE: pulmonary embolism

Major bleeding coding algorithm available at:

Fang MC, Go AS, Chang Y, et al. A new risk scheme to predict warfarin-associated hemorrhage: The ATRIA (anticoagulation and risk factors in atrial fibrillation) study. *J Am Coll Cardiol* 2011;58(4):395-401.

**Appendix B.** Standardized Differences Relative to Warfarin Group (Significant value >0.10)

| | LMWH | Filter | Filter + Anticoagulation | None |
|---|---|---|---|---|
| Age (continuous) | 0.1213 | 0.1058 | 0.0035 | 0.0094 |
| Age (categorical) | 0.1070 | 0.0424 | 0.0661 | 0.0147 |
| Plan type | 0.1294 | 0.0967 | 0.0417 | 0.0019 |
| Region | 0.0849 | 0.0749 | 0.0323 | 0.0080 |
| Gender | 0.0784 | 0.0414 | 0.0162 | 0.0072 |
| Index event | 0.0323 | 0.1906 | 0.0618 | 0.0097 |
| Race | 0.1341 | 0.0577 | 0.0452 | 0.0101 |
| CCI (categorical) | 0.1088 | 0.1639 | 0.0630 | 0.0042 |
| Leukocytosis | 0.0471 | 0.0014 | 0.0689 | 0.0108 |
| Leukocytopenia | 0.0038 | 0.0087 | 0.0043 | 0.0063 |
| Thrombocytopenia | 0.0638 | 0.0175 | 0.0132 | 0.0113 |
| Thrombocytosis | 0.0072 | 0.0468 | 0.0379 | 0.0034 |
| Hypocoagulatory disorder | 0.0160 | 0.0724 | 0.0450 | 0.0046 |
| Anemia | 0.0349 | 0.0239 | 0.0609 | 0.0100 |
| Liver dysfunction | 0.0078 | 0.0325 | 0.0231 | 0.0047 |
| Renal dysfunction | 0.0635 | 0.0707 | 0.0271 | 0.0136 |
| Hypertension | 0.0357 | 0.0424 | 0.0634 | 0.0041 |
| Prior bleed | 0.0027 | 0.0360 | 0.0206 | 0.0023 |
| Obesity | 0.0030 | 0.0175 | 0.0046 | 0.0041 |
| Treatment timing | 0.0608 | 0.0706 | 0.0339 | 0.0051 |
| **Tumor Site** | | | | |
| Oral | 0.0265 | 0.0173 | 0.0126 | 0.0050 |
| Stomach | 0.0227 | 0.0218 | 0.0068 | 0.0040 |
| Colon | 0.0260 | 0.0547 | 0.0175 | 0.0047 |
| Liver | 0.0053 | 0.0423 | 0.0004 | 0.0079 |
| Pancreas | 0.0198 | 0.0146 | 0.0335 | 0.0001 |
| Lung | 0.0304 | 0.0960 | 0.0346 | 0.0068 |
| Breast | 0.0092 | 0.0249 | 0.0117 | 0.0027 |
| Melanoma | 0.0420 | 0.0699 | 0.0460 | 0.0030 |
| Uterine | 0.0420 | 0.0699 | 0.0460 | 0.0030 |
| Cervix | 0.0003 | 0.0217 | 0.0037 | 0.0109 |
| Ovarian | 0.0226 | 0.0098 | 0.0220 | 0.0028 |
| Prostate | 0.0477 | 0.0554 | 0.0234 | 0.0088 |
| Bladder | 0.0016 | 0.0317 | 0.0126 | 0.0021 |
| Renal | 0.0635 | 0.0707 | 0.0271 | 0.0136 |
| Brain | 0.0503 | 0.0473 | 0.0014 | 0.0150 |
| Thyroid | 0.0950 | 0.0064 | 0.0074 | 0.0039 |
| Lymphoma | 0.0106 | 0.0777 | 0.0050 | 0.0015 |
| Myeloma | 0.0201 | 0.1132 | 0.0160 | 0.0023 |
| Metastasis | 0.0166 | 0.0806 | 0.0685 | 0.0020 |
| Myocardial infarction | 0.0244 | 0.0653 | 0.0309 | 0.0050 |
| Congestive heart failure | 0.0531 | 0.0904 | 0.0288 | 0.0051 |
| Peripheral vascular disease | 0.0624 | 0.1103 | 0.0303 | 0.0023 |
| Cerebrovascular disease | 0.0109 | 0.0974 | 0.0285 | 0.0136 |

**Appendix B.** Standardized Differences Relative to Warfarin Group (Significant value >0.10) - cont'd

| | LMWH | Filter | Filter + Anticoagulation | None |
|---|---|---|---|---|
| Dementia | 0.0044 | 0.0586 | 0.0376 | 0.0085 |
| Chronic pulmonary disease | 0.0530 | 0.0432 | 0.0054 | 0.0006 |
| Rheumatic disease | 0.0453 | 0.1112 | 0.0202 | 0.0059 |
| Peptic ulcer disease | 0.0349 | 0.0039 | 0.0095 | 0.0064 |
| Diabetes w/o complications | 0.0022 | 0.0536 | 0.0579 | 0.0079 |
| Diabetes w/ complications | 0.0252 | 0.0517 | 0.0212 | 0.0004 |
| Paraplegia/hemiplegia | 0.0751 | 0.0258 | 0.0373 | 0.0102 |
| Depression | 0.0706 | 0.0236 | 0.0167 | 0.0013 |
| Skin ulcers/cellulitis | 0.0677 | 0.0894 | 0.0488 | 0.0012 |
